# A Topology Control Strategy with Reliability Assurance for Satellite Cluster Networks in Earth Observation

**DOI:** 10.3390/s17030445

**Published:** 2017-02-23

**Authors:** Qing Chen, Jinxiu Zhang, Ze Hu

**Affiliations:** 1 Research Center on Satellite Technology, Harbin Institute of Technology, Harbin 15001, China; 14B918054@hit.edu.cn; 2 School of Computer Science and Technology, Harbin Institute of Technology, Harbin 150001, China; huze@ftcl.hit.edu.cn

**Keywords:** topology control strategy, reliability assurance, satellite mobility, satellite cluster networks, earth observation

## Abstract

This article investigates the dynamic topology control problem of satellite cluster networks (SCNs) in Earth observation (EO) missions by applying a novel metric of stability for inter-satellite links (ISLs). The properties of the periodicity and predictability of satellites’ relative position are involved in the link cost metric which is to give a selection criterion for choosing the most reliable data routing paths. Also, a cooperative work model with reliability is proposed for the situation of emergency EO missions. Based on the link cost metric and the proposed reliability model, a reliability assurance topology control algorithm and its corresponding dynamic topology control (RAT) strategy are established to maximize the stability of data transmission in the SCNs. The SCNs scenario is tested through some numeric simulations of the topology stability of average topology lifetime and average packet loss rate. Simulation results show that the proposed reliable strategy applied in SCNs significantly improves the data transmission performance and prolongs the average topology lifetime.

## 1. Introduction

Earth observation (EO) is the information gathering of Earth’s physical, chemical, and biological systems by the use of remote sensing technologies. EO with satellites has been implemented in many different areas, such as weather forecasting, natural disaster monitoring, natural resources management, land-use change measurement, and others [1,2]. However, when facing some emergency services, it is a challenge for current EO satellites to complete the target information acquisition and transmission in a short time [3], for the actual performance of task execution in EO missions with traditional satellites is limited by several factors, like short communication windows between satellites and ground stations, low data transmitting rates from satellites to ground, and large amounts of image data [4]. It means that the contemporary work mode of EO satellites is difficult to meet the requirements of the complex future EO situations, especially in some emergency situations. Fortunately, with the great improvement of satellite technology in recent years [5,6], especially in areas of nano-satellites, low-cost rockets, and launch methods, satellite clusters, as a new space system for future space missions, has attracted a lot of attention.

Similar to mobile wireless sensor networks (MWSNs) [7], satellite cluster networks (SCNs) is a self-organizing distributed satellite system, in which several cooperatively-working satellites work together to accomplish the complex space mission through wireless inter-satellites links [8]. Though this conception of SCNs with cooperative work has been advanced long before, it has been ignored because of the limitation of high cost of satellites’ research and development. In 1991, Ganz et al. [9,10] analyzed the system load and traffic distribution of SCNs based on an exact Markov modeling, and established a mathematical model of network capacity. In recent years, due to the development of satellites technologies, like miniaturization and highly integration, researchers re-focused their minds on the research of SCNs. Feihong Dong et al. [11] took the SCNs with fifteen satellites in geosynchronous orbit (GSO) as the research object, figuring out that the hybrid architecture realizes a stunning trade-off between the network efficiency and robustness. Zhong et al. [12] analyzed the characteristics of the distributed satellite cluster network and proposed a power control approach based on presetting prediction. For problems in task scheduling, a predictive-reactive scheduling model has been proposed using the hybrid of periodic task scheduling and dynamic adjustment [13]. Though some technical problems in SCNs have been widely researched, the technique of topology control strategy in SCNs is still immature and there are only a few studies focused on it.

Topology control strategies are included in network management techniques. Because of the unstable inter-satellites links (ISLs) in SCNs and the possible data transmitting errors among satellites, the main goal of topology control in SCNs is to achieve the most stable data routing paths for the cooperative-working satellites. However, topology control strategy, as one of the key technologies in SCNs, has seldom been studied in recent years [14,15]. In 2016, Zhang et al. [16] proposed logical *k*-connected topology control algorithms, including both the purely centralized and the purely distributed, but it mainly focuses on a hierarchical integrated network which include not only SCNs, also the unmanned aerial vehicles (UAVs) and WSNs. And the algorithms take the satellites in one cluster as a node, and do not establish the inner topology of the cluster.

In other research areas, UAVs and MWSNs, network topology control techniques for mobile self-organization networks have been deeply researched [17,18,19]. For example, the neighbors graph (Neigh) constructs the topology with probability [20], the low interference forest establisher (LIFE) uses the value of nodes covered by link to reestablish the minimum spanning tree (MST) [21], and the fault-tolerant local s panning subgraph (FLSS) [22] guarantees the capacity of fault tolerance. Zhang et al. [23] proposed a distributed battery recovery effect aware connected dominating set constructing algorithm (BRE-CDS) to prolong the network lifetime of wireless sensor networks. However, most topology control algorithms in these fields are established in two-dimensional (2D) planes while SCNs is a kind of dynamic networks working in three-dimensional (3D) space. In [24], Wang et al. extended several 2D geometric topologies to 3D case and proposed a new 3D flexible Yao-based graph (FlYG) algorithms for sensor networks. Nevertheless, FlYG only considered the static nodes, not involving dynamic situations, and is not appropriate for SCNs with dynamic relative movement [25,26].

Considering the deficiency of present research, this article focuses on the problem of topology control strategy in SCNs when working in some emergency EO missions. First, we describe the SCNs with time snapshot by using the Graph Theory, and give the definition of SCNs by considering its bounded relative motion. Then, we suppose an emergency situation with cooperative-working SCNs in EO missions and give its mathematical model. Because of the network properties of relative motion, unstable and periodic changes in topology, SCNs requires reliable topology and ISLs to ensure the stability of data transmission. Thus, based on the equation of relative motion of satellites [27], we propose a novel link cost metric using the properties of prediction and periodicity of satellites movement. Also, a general reliability model of SCNs is analyzed to give a selection criterion of choosing suitable ISLs in the proposed topology control strategy. Briefly, the proposed topology control strategy, based on the novel link metric with reliability and the selection criterion, consists of three phases: (i) Satellites in SCNs analyze the network links’ connections and obtains the reliability-optimized topology matrix; (ii) The topology matrix is distributed to every satellite in SCNs; (iii) In each topology updating period, SCNs changes the link connections according to the topology matrix. The validity of the proposed strategy will be demonstrated by using comprehensive experiments.

In conclusion, the main contributions of this paper, given the state-of-the-art research work, are mainly summarized in the following three aspects:

- A cooperative work model in SCNs with emergency EO missions is proposed to give an application case when facing emergency situations. The large scale, complex and dynamic SCNs is decoupled into three kinds of satellites through different working modes, which make the topology control of SCNs easier.
- A novel link cost metric of reliability is proposed to give the selection criterion for choosing the most reliable data routing paths. Through the laws of satellites relative motion, the periodicity and predictability of satellites’ relative position in SCNs are involved in the link cost metric to enhance each chosen ISLs’ reliability.
- A topology control strategy with reliability assurance is proposed to solve the problem of topology management in the dynamic and unstable SCNs. Through some numeric simulations, the proposed reliable strategy has been tested in term of effectiveness of improving data transmission performance and extending average topology lifetime.

The rest of this paper is organized as follows: Section 2 gives a network description about the satellite cluster with EO missions. In Section 3, the link cost metric with reliability assurance is discussed. Section 4 presents a topology control strategy for a satellite cluster network with an EO mission. An example and simulation to furtherly illustrate the performance of the proposed topology is shown in Section 5. Finally, in Section 6, the conclusion is summarized.

## 2. Network Description

SCNs is a type of new distributed wireless space system, in which most space missions work cooperatively through ISLs among the satellites. In this section, this paper first presents a proper network description and some definitions and assumptions for SCNs, then puts forward a mathematical model of cooperative work for EO missions.

### 2.1. Satellite Cluster Network

**Figure 1:**
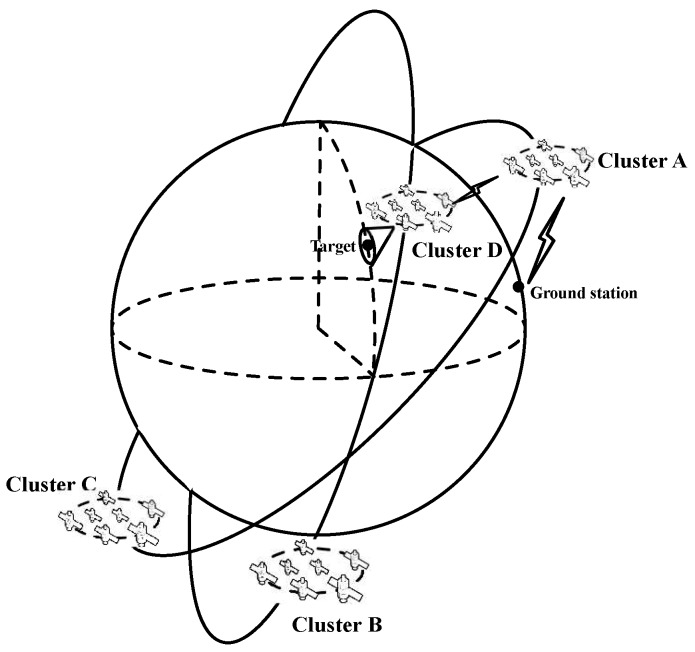
Illustration of satellite cluster networks (SCNs) with multi-clusters.

**Figure 2:**
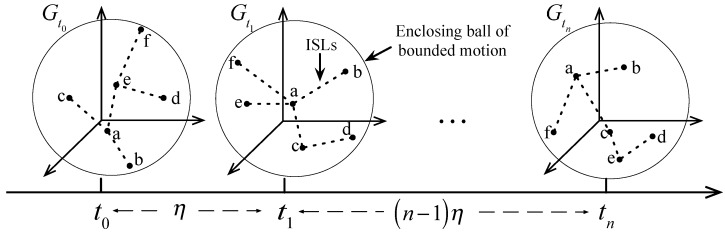
An example of topology changing varying with time.

Similar to MWSNs [28,29], this paper first supposes that there are *N* satellites in the SCNs. Then, the network can be described as $G=(V_{\left[N,3\right]},E_{\left[N,N\right]})$ using the Graph Theory, where $V_{\left[N,3\right]}$ denotes the vertex of nodes positions in an Earth-centered inertial reference frame (ECI) and $E_{\left[N,N\right]}$ is the set of undirected ISLs among satellites. Let the network be composed of *u* clusters, and each cluster has $b_{i}$ satellites and $c_{i}$ links, i.e., $N=\sum_{i=1}^{u}b_{i}$. Figure 1 shows the structure of SCNs with four clusters and the cluster A and D run cooperatively to accomplish EO missions (satellites in cluster D are responsible for target detection and clusters A transmits the data of images to the ground station). Moreover, due to satellites in SCNs moves around each other varying the time, the network topology using snapshot can be represented as: $G=\left\{G_{t_{0}},\cdots ,G_{t},\cdots \right\}=\left\{G_{t_{n}}|t_{n}=t_{0}+n\eta \right\}$, where, $t_{0}$ denotes the initial time of network topology, *η* is the time interval of every-two snapshots, and *n* denotes any positive integer. For an example, Figure 2 illustrates the topology snapshots of dynamic time varying network, where nodes a to f represent the satellites running with each other in a known range [30].

**Definition** **1.** (Satellite cluster networks, SCNs) If a number of satellites on orbit run around each other in a certain range and can establish ISLs with others, a area-network consisting of these satellites is a sub-SCNs, like cluster A in Figure 1, and several sub-SCNs construct the SCNs.

**Remark** **1.** In astrodynamics, as the satellites in SCNs fly around each other and also orbit the same central body earth, it signifies that they have the same orbit period and the uniform orbit altitude or semi-major axis [30].

The following content in this paper focuses on the dynamic topology control strategy in EO missions in SCNs and also makes the assumptions below: (1) during the process of inter-satellite communication, each satellite has the same maximum transmission range, and always keeps the maximum transmitting power if it is active; (2) antennas on satellites are in an ideal omni-directional pattern; (3) communication protocols on each satellite have ideal MAC layer, which means any two satellites can establish the communication link only if they are in the communication range; (4) each satellite using wireless links in SCNs shares with others its navigation information periodically [31], including position and velocity in ECI; (5) every satellite in SCNs runs in a circular orbit without control and ignores any perturbations from celestial bodies except earth.

### 2.2. EO Missions on Satellite Cluster Network

According to the usual workflow in Earth observation [32,33], after the step of mission planning, the next is taking images of targets using the payloads. Once the target area has been detected and covered, the satellite begins the procedure of transmitting target images to the ground stations. Even though, in some cases, satellites can work in both states of image-taking and ground communication, the download bandwidth is very low due to the limited energy onboard and vast distance between the ground and satellite. Thus, it may take a long time from gathering images to accomplishing image-sending. To resolve this problem, this paper puts forward a cooperative work pattern in SCNs.

**Figure 3:**
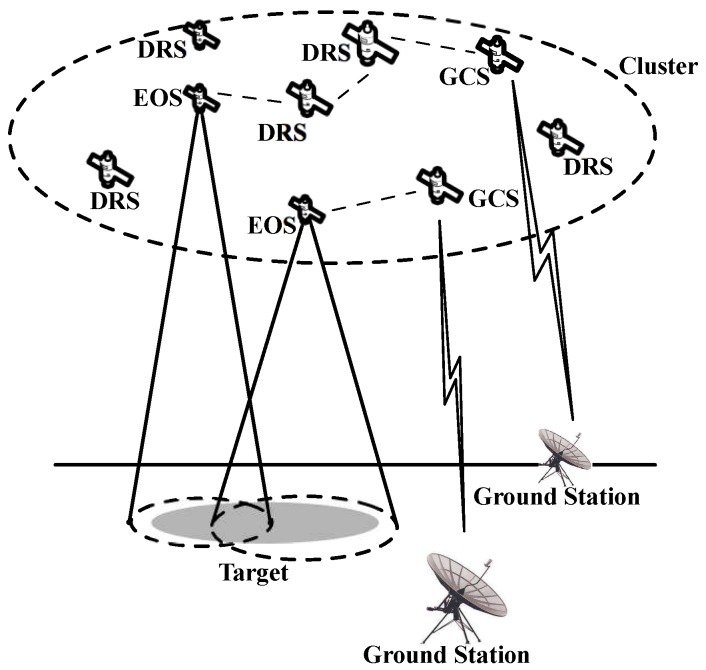
Illustration of cooperative work in SCNs.

According to different payload requirements when observing the Earth, let satellites in SCNs be divided into three categories: Earth observation satellites (EOS), ground-station communication satellites (GSC), and data routing satellites (DRS), i.e., $V_{\left[N,3\right]}=V_{EOS}+V_{GSC}+V_{DRS}$. EOS are the satellites having EO cameras and play the role of taking images of the ground targets; GSC should have the ability of high-speed data transmission and are responsible for sending these data to ground stations via satellite-ground links of high bandwidth; and the rest satellites are categorized as DRS, which undertake the task of image data relaying, to transmit the image data from EOS to GSC. For example, as shown in Figure 3, two EOS work cooperatively to accomplish the target imaging and, at the same time, send these data to the ground stations via several DRS and GSC. In this above cooperative work pattern, it not only greatly extends the functionality of the SCN by working as a team, but also makes possible the transmission of large amounts of image data to the ground, which is usually strongly demanded in some emergency cases [34].

In SCNs, this paper respectively indicates any satellite in EOS, DRS, and GSC by using $s_{i}$, $r_{i}$ and $g_{i}$, i.e., $s_{i}\in V_{EOS}$, $r_{i}\in V_{DRS}$ and $g_{i}\in V_{GSC}$. *n* and *m* denote the number of satellites in EOS and GSC separately. Additionally, *l* is the number of DRS with data routing requirements in one specific topology snapshot, and $l^{\prime }$ shows the rest of the DRS not getting involved in routing tasks during one snapshot. Based on the states of whether participating in cooperative work, the topology of SCNs can be indicated with the gathering of the working sub-graph $G_{t}^{\prime }$ and non-working sub-graph $G_{t}^{\prime \prime }$, i.e., $G_{t}=G_{t}^{\prime }+G_{t}^{\prime \prime }=\left(V_{t}^{\prime },E_{t}^{\prime }\right)+\left(V_{t}^{\prime \prime },E_{t}^{\prime \prime }\right)$. The position vertex of satellites in the cluster satisfy the following relations:

(1) $$\begin{array}{ccc} V_{t} & = & V_{t}^{\prime }+V_{t}^{\prime \prime }\\ & = & V\left(s_{1},\cdots ,s_{n},r_{1},\cdots ,r_{l},g_{1},\cdots g_{m}\right)\\ & & +V\left(r_{l+1},\cdots ,r_{l+l^{\prime }}\right) \end{array}$$

where:

(2) $$\left\{\begin{array}{c} n+m+l+l^{\prime }=N\\ V_{EOS}=\left\{s_{1},\cdots ,s_{n}\right\}\\ V_{DRS}=\left\{r_{1},\cdots ,r_{l},r_{l+1},\cdots ,r_{l+l^{\prime }}\right\}\\ V_{GSC}=\left\{g_{1},\cdots g_{m}\right\} \end{array}\right.$$

The equations above describe the position relations $V_{t}$ of one topology snapshot, and the subsequent content will discuss the edge relations $E_{t}$ in detail, with consideration of orbits’ prediction and periodicity.

## 3. Link Cost Metric With Reliability Assurance

In EO missions, in order to ensure the stability of data transmission, it puts forward high requirements for topology and ISLs reliability, due to satellites’ relative movements, unstable and periodic changes in topology, and also the data transmitting errors of satellites navigation information. This section focuses on the topology reliability and link cost metric with consideration of the network properties of prediction and periodicity.

### 3.1. Satellite Mobility

**Figure 4:**
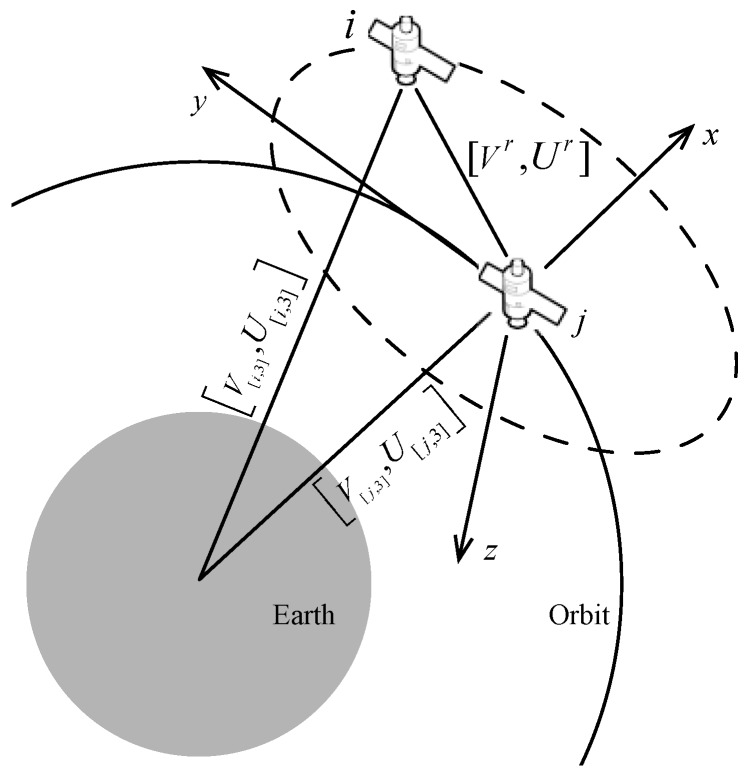
Illustration of transforming position and velocity in ECI to the relative coordinate system of satellite *j*.

First, on the basis of Assumption (4) in Section 2, each satellite in the SCN has a network position matrix $V_{\left[N,3\right]}$ and its corresponding velocity matrix $U_{\left[N,3\right]}$ in ECI at time *t*. Then, in order to describe the mobility of satellites in detail, it is necessary to change positions and velocity in ECI to a relative coordinate system and obtain the relative position matrix $V^{r}$ and relative velocity matrix $U^{r}$, as shown in Figure 4, where the relative position and velocity matrix $\left[V^{r},U^{r}\right]$ meet the following equation:

(3) $${\left[V^{r},U^{r}\right]}^{\prime }=\left({\left[V_{\left[i,3\right]}^{r},U_{\left[i,3\right]}^{r}\right]}^{\prime }-{\left[V_{\left[j,3\right]}^{r},U_{\left[j,3\right]}^{r}\right]}^{\prime }\right)A$$

where *A* is the coordinate transformation matrix [35].

Let the time of $V^{r}$ and $U^{r}$ be the initial time, i.e., t=0, $V^{r}=\left[x_{0},y_{0},z_{0}\right]$, $U^{r}=\left[{\dot{x}}_{0},{\dot{y}}_{0},{\dot{z}}_{0}\right]$. According to Assumption (5), satellites run in a near-circular orbit and ignore any disturbance. Then, from the literature [27], for any two satellites, *i* and *j*, the relative position from *i* to *j* meets the following equations:

(4) $$\left\{\begin{array}{c} x\left(t\right)=-\left(3x_{0}+2{\dot{y}}_{0}/\omega \right)\cos\omega t+{\dot{x}}_{0}/\omega \sin\omega t+2\left(2x_{0}+2{\dot{y}}_{0}/\omega \right)\\ y\left(t\right)=2\left(3x_{0}+2{\dot{y}}_{0}/\omega \right)\sin\omega t+2{\dot{x}}_{0}/\omega \cos\omega t+y_{0}-2{\dot{x}}_{0}/\omega -3\left({\dot{y}}_{0}+2\omega x_{0}\right)t\\ z\left(t\right)=z_{0}\cos\omega t+{\dot{z}}_{0}/\omega \sin\omega t \end{array}\right.$$

where:

(5) $$\omega =\sqrt{\mu a_{j}\left(1-e_{j}\right)}/\left|V_{\left[j,3\right]}\right|$$

and *ω* is the orbit instantaneous angular rate of *j*, $\left[x_{0}\;y_{0}\;z_{0}\;{\dot{x}}_{0}\;{\dot{y}}_{0}\;{\dot{z}}_{0}\right]$ is the initial relative position and velocity for *i* to *j*, $|V_{\left[j,3\right]}|=\sqrt{x_{j}^{2}+y_{j}^{2}+z_{j}^{2}}$ is the geocentric distance of *j*, *μ* is the standard gravitational parameter of the Earth, a constant value of $3.986\times 10^{14}\;m^{3}/s^{2}$, and $a_{j}$, $e_{j}$ is the orbit’s semi-major axis and eccentricity of *j*, respectively.

Additionally, from the Definition 1 of SCNs, satellites in SCNs must runs with others in a bounded range, and this means that the equations above do not have the offset of time [27], i.e., $-3\left({\dot{y}}_{0}+2\omega x_{0}\right)t=0\Rightarrow {\dot{y}}_{0}=-2\omega x_{0}$. Then, Equation (4) can be simplified as:

(6) $$\left\{\begin{array}{c} x\left(t\right)=-\left(3x_{0}+2{\dot{y}}_{0}/\omega \right)\cos\omega t+{\dot{x}}_{0}/\omega \sin\omega t\\ y\left(t\right)=2\left(3x_{0}+2{\dot{y}}_{0}/\omega \right)\sin\omega t+2{\dot{x}}_{0}/\omega \cos\omega t+y_{0}-2{\dot{x}}_{0}/\omega \\ z\left(t\right)=z_{0}\cos\omega t+{\dot{z}}_{0}/\omega \sin\omega t \end{array}\right.$$

Thus, the relative motion of every two satellites in the SCN can be defined as Equation (6), which can be used to predict any other satellites’ positions, since each satellite can obtain all others’ positions through cyclic navigation information sharing.

Meanwhile, by the analysis of Equation (6), the period *T* of *j* orbiting around can be calculated as:

(7) $$\begin{array}{cc} T_{ij} & =2\pi /\omega \\ & =2\pi |V_{\left[j,3\right]}|/\sqrt{\mu a_{j}\left(1-e_{j}^{2}\right)} \end{array}$$

In Equation (7), both $a_{j}$ and $e_{j}$ can be regarded as constants for the reference satellite *j*, since the satellites orbit without any orbit control and any perturbations. Thus, the period *T* of relative motion is fixed for every two satellites, and also in the relative coordinate system of satellite *j*, as other satellites’ periods of flying around *j* are equivalent.

### 3.2. Reliability Model for Network With EO Missions

In SCNs, the shared navigation information has some measuring errors, which cause the deviations between actual values of satellite position and the measured values. Additionally, this also leads to the differences that some essential data are calculated by math recursion on the orbit dynamic model. The deviations and differences create the state of wireless links in some uncertain situations, such as link interruption, lower bandwidth, etc.

In this paper, it is assumed that the probability of every working link in normal work is r(0<r<1) and the effective working time per period for each satellite is *τ*. Then, each link’s reliability model can be represented as:

(8) $$Y=r\tau T^{-1}$$

which represents the probability of normal work per period.

According to the proposed cooperative work pattern in Section 2, let the reliability of the communication path $\left(s_{j}\to t_{j}\right)$ be $Y_{ij}$, then, it satisfies the following equations:

(9) $$Y_{ij}=\underset{\alpha_{ij}}{\underset{︸}{Y_{1}\cdots Y_{e}\cdots }}=r^{\alpha_{ij}}\prod_{e=1}^{\alpha_{ij}}\left(\tau_{e}T_{e}^{-1}\right)\;\;$$

where:

(10) $$\left\{\begin{array}{c} 1\leq a_{ij}\leq N-1\\ 1\leq i\leq n\\ 1\leq j\leq m \end{array}\right.$$

where $a_{ij}$ is the number of links on the path $\left(s_{j}\to t_{j}\right)$, i.e., the hop number of data routing, *n* and *m* denotes the number of satellites in EOS and GSC, respectively.

In any EO mission, it can be regarded that the state of the network operating normally means all image data produced from EOS can be transmitted to GSC successfully and the construction of each sub-network $s_{i}\to \left(t_{1},t_{2},\cdots ,t_{m}\right)$ of EOS to GSC are independent events. According to the independence of probability theory, let the probability of the normal work state be *P*, and it meets the following relations:

(11) $$\begin{array}{ccc} P\left\{\left(s_{1},s_{2},\cdots ,s_{n}\right)\to \left(t_{1},t_{2},\cdots ,t_{m}\right)\right\} & = & P\left\{\left(s_{1}\to \left(t_{1},t_{2},\cdots ,t_{m}\right)\right)⋂\cdots ⋂\left(s_{n}\to \left(t_{1},t_{2},\cdots ,t_{m}\right)\right)\right\}\\ & = & P\left(s_{1}\to \left(t_{1},t_{2},\cdots ,t_{m}\right)\right)⋂\cdots ⋂P\left(s_{n}\to \left(t_{1},t_{2},\cdots ,t_{m}\right)\right)\\ & = & \prod_{i=1}^{n}P_{i} \end{array}$$

Thus, combined with the equation above, to guarantee stable image data transmission, on behalf of the independence of $P_{i}$, network reliability meets the constraints below:

(12) $$\max P=\max\prod_{i=1}^{n}P_{i}=\prod_{i=1}^{n}\max P_{i}$$

**Figure 5:**
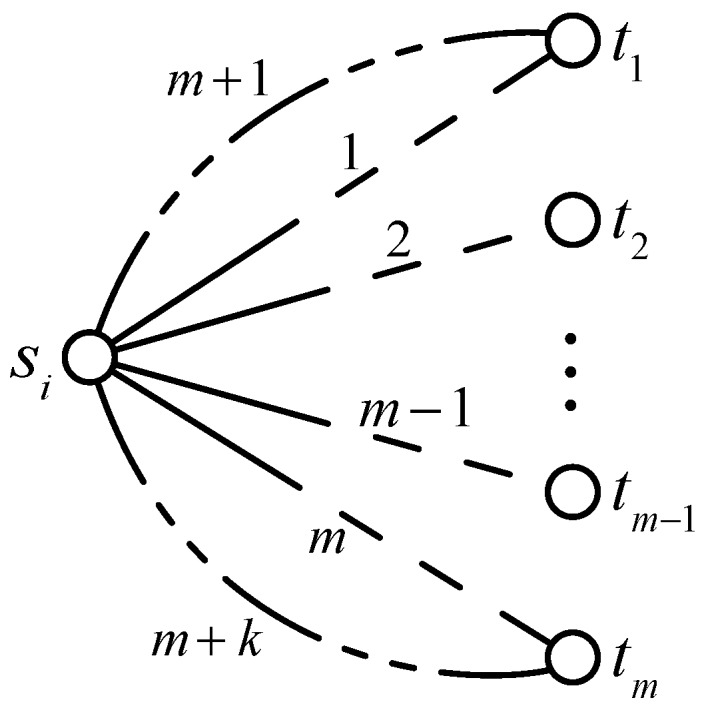
Topology configuration with the highest reliability (*m* shortest paths, *k* redundant paths).

Equation (12) shows that the optimal reliability of the whole network topology requires each sub-topology’s reliability to be optimal. Additionally, it is obvious that when the sub-topology is designed as the configuration in Figure 5, i.e., all paths are in parallel, the reliability is the best.

Then, Equation (11) can be changed as follows:

(13) $$\begin{array}{ccc} \max P & = & \prod_{i=1}^{n}\max\left[1-\prod_{j=1}^{m}\left(1-Y_{ij}\right)\prod_{q=1}^{k}\left(1-Y_{iq}\right)\right]\\ & = & \prod_{i=1}^{n}\max\left\{1-\prod_{j=1}^{m}\left[1-r^{\alpha_{ij}}\prod_{e=1}^{\alpha_{ij}}\left(\tau_{e}T_{e}^{-1}\right)\right]\prod_{q=1}^{k}\left[1-r^{\alpha_{iq}}\prod_{e=1}^{\alpha_{iq}}\left(\tau_{e}T_{e}^{-1}\right)\right]\right\} \end{array}$$

s.t.:

(14) $$\left\{\begin{array}{c} 1\leq a_{ij}\leq N-1\\ 1\leq a_{iq}\leq N-1\\ 1\leq Y_{j},Y_{q},r\leq 1 \end{array}\right.$$

where, *k* is the number of redundant paths of the sub-topology $s_{i}\to \left(t_{1},t_{2},\cdots ,t_{m}\right)$. In Equation (14), when $a_{j}=1$, $a_{k}=2$, it can be proved that the network has the best reliability assurance and the probability of normal work is $\prod_{i=1}^{n}\left[1-\prod_{j=1}^{m}\left(1-r\tau_{j}T_{j}^{-1}\right)\prod_{q=1}^{k}\left(1-r\tau_{q}T_{q}^{-1}\right)\right]$. However, under the actual work conditions, limited by the maximum communication range, it is difficult to make $a_{j}=1$, i.e., transmitting data from EOS to GSC directly.

Furthermore, because all of the variables in Equation (13) are not correlative, it can be further simplified as:

(15) $$\max P=\left\{\begin{array}{c} \max k\\ \min\alpha_{ij}\\ \min\alpha_{iq} \end{array}\right.$$

Finally, from the above equation, it denotes that for better network reliability, there exist two possible methods: reducing the number of each path’s hop, or increasing the redundant paths’ number.

### 3.3. Link’s Stability Model

The main purpose of this paper is to research the methods of improving the satellite network reliability. A usual method of this is replacing the link’s metric of distance with its reliability as the link stability metric. This part discusses the satellite cluster’s link cost metric with the links’ reliability and satellite dynamic model.

From Equation (8), each link’s reliability can be represented as $Y=r\tau T^{-1}$, and through the min-max normalization, the reliability is changed to:

(16) $$\begin{array}{ccc} \chi_{ij} & = & \left(Y_{ij}-Y_{\min}\right){\left(Y_{\max}-Y_{\min}\right)}^{-1}\\ & = & \left[{\left(\tau T^{-1}\right)}_{ij}-{\left(\tau T^{-1}\right)}_{\min}\right]{\left[{\left(\tau T^{-1}\right)}_{\max}-{\left(\tau T^{-1}\right)}_{\min}\right]}^{-1} \end{array}$$

The period time *T* can be calculated by Equation (7), and the effective working time *τ* per period can also be calculated through the following equation:

(17) $$\tau =\left\{t|x^{2}\left(t\right)+y^{2}\left(t\right)+z^{2}\left(t\right)\leq R^{2},\;0\leq t\leq T\right\}$$

Thus, this paper sets Equation (16) to be the link cost metric. Finally, through the Equations (6), (16) and (17), all of the possible links’ cost matrices $\left[\chi_{ij}\right]$ can be obtained with reliability.

## 4. Reliability Assurance Topology Control Strategy

In the previous section, this paper proposed some possible methods (e.g., increasing the number of redundant paths) to make the network more reliable and improve the link cost metric’s reliability. In this section, this paper tries to construct a topology control strategy for a satellite cluster with EO missions by using the results discussed above.

Considering that a satellite’s approximate position can be predicted with an orbit dynamic model, this paper uses the centralized topology control strategy in general. This means one satellite in the cluster periodically calculates all of the possible network links and then distributes the results to others.

The process of the topology control strategy in this paper includes two main parts: topology matrix calculation and its distribution. Topology matrix calculation means one of the DRS analyzes the network links’ connections and obtains the reliability-optimized topology. Then, the procedure of distribution is that the satellite with the links’ relations sends it to the others. After that, in one topology updating period, the network changes the link connections according to the topology matrix.

The topology control strategy with reliability assurance in the satellite cluster can be regarded as the following three steps:

Step1: Topology Matrix Calculation

In this step, we present a centralized algorithm for one satellite cluster with EO missions. One of the DRS will calculate the links for all. This step mainly includes three sub-steps: (1) calculating the paths for sending image data from any of the EOS $s_{i}$ to the GSC $\left(g_{1},g_{2},\cdots ,g_{m}\right)$ to obtain $p_{i}=\left\{s_{i}\to \left(g_{1},g_{2},\cdots ,g_{m}\right)\right\}$, including the abundant paths; (2) repeating Sub-step (1) to obtain sub-topology $G_{sg}=\left\{p_{1},\cdots ,p_{m}\right\}$ for all EOS to GSC; and (3) connecting the isolated satellites to the nearest and connected satellite to obtain the final topology $G_{t}$. The centralized topology control algorithm is described in Algorithm 1, where *s*, *g* and *r* represent the position matrix of EOS, GSC, and DRS at time *t*, respectively.
**Algorithm 1** Reliability-Assured Topology (RAT)**Input:**
*s*, *g*, *r* and $\left[\chi_{ij}\right]$**Output:**
$G_{t}=\left(V_{t},E_{t}\right)$1:% Sub-step (1,2)2:$V_{t}=\left(s,g,r\right)$, $E_{t}=\emptyset$, n=length(s), m=length(g)3:**for**
i=1 to *n*
**do**4: **for**
j=1 to *m*
**do**5:  $p_{s_{i}g_{j}}=ShortestPath\left(s_{i},g_{j}\right)$ in $s_{i},g_{j},r$6: **end**
**for**7: $p_{s_{i}g}=\left\{p_{s_{1}g_{1}},\cdots ,p_{s_{1}g_{m}}\right\}$8: Sort $p_{s_{i}g}$ into non-decreasing order by the path cost with $\left[\chi_{ij}\right]$9: Let $r_{1},\cdots ,r_{m}$ be the sets of DRS on the paths $p_{1},\cdots ,p_{m}$, respectively10: **for**
k=2 to *m*
**do**11:  **if**
$r_{k}\cap \left(r_{1}\cup \cdots \cup r_{k-1}\right)\neq \emptyset$
**then**12:   $p_{k}^{\prime }=ShortestPath\left(s_{i},g_{k}\right)$ in $s_{i},g_{k},r/\left(r_{1}\cup \cdots \cup r_{k-1}\right)$13:   **if**
$p_{k}^{\prime }=\emptyset$
**then**14:    $p_{k}^{\prime }=p_{k}$15:   **end**
**if**16:  **else**17:   $p_{k}^{\prime }=p_{k}$18:  **end**
**if**19: **end**
**for**20: $p_{s_{i}g}^{\prime }=\left\{p_{1}^{\prime },\cdots ,p_{m}^{\prime }\right\}$21:**end**
**for**22:$G_{t}=\left\{p_{s_{1}g}^{\prime },\cdots ,p_{s_{n}g}^{\prime }\right\}$23:%Sub-step (3)24:**if**
$G_{t}$ exists isolated satellite **then**25: **for** any isolated satellite **do**26:  Connect the isolated satellite to the nearest and connected satellite and get $G_{t}$27: **end**
**for**28:**end**
**if**29:**return**
$G_{t}$

**Theorem** **1.** If the SCN’s initial topology without any topology control is connected, the topology after using the RAT algorithm is also connected.

**Proof of Theorem** **1.** Let us assume the SCN’s topology $G_{t}$ with RAT is not connected, i.e., the network can be divided into several unconnected sub-topologies, like $G_{t}=G_{0}+\cdots +G_{s}$. As the initial topology is connected, this means the RAT algorithm of Sub-step (3) cannot change the connectivity by just connecting the isolated satellite to the nearest and connected satellite. Then, the reason for making the topology unconnected is from the Sub-step (1) or (2), i.e., $G_{t}=\left\{p_{s_{1}g}^{\prime },\cdots ,p_{s_{n}g}^{\prime }\right\}$ is unconnected and can be changed into $G_{t}=G_{0}^{\prime }+\cdots +G_{s}^{\prime }$, where $G_{0}^{\prime },\cdots ,G_{s}^{\prime }$ have no intersections. However, all of the paths established from Sub-steps (1) and (2) have the same intersections of satellites, like GSC. Then, there exists a contradiction and the assumption is not valid. ☐

Step 2: Topology Recursion

Based on the topology snapshot interval *η* and the satellites initial positions $V_{0}$, the position matrix $V_{t}=\left\{V_{t}|t=t_{0}+n\eta \right\}$ in the next snapshot can be calculated by using the satellite dynamic model and the Runge-Kutta algorithm [36]. Then, by repeating Step 1, the strategy obtains the topologies of the satellite cluster at every time of snapshot, $G_{t}=\left\{G_{t}|t=t_{0}+n\eta \right\}$.

Step 3: Topology Distribution

The satellite of calculation determines the MST topology $G_{MST}$ of the satellite cluster, and sends the data of topology $G_{t}=\left\{G_{t}|t=t_{0}+n\eta \right\}$ to all ofthe other satellites through $G_{MST}$.

Step 4: Strategy Execution

From the start time $t_{0}$ of EO missions and the next moments $t=t_{0}+n\eta$ of topology switching, every satellite changes the adjacent satellite links according to $G_{t}$.

After applying the above four steps, this paper finally obtains a proper topology for the SCN with EO missions.

## 5. Example and Discussion

In this section, this paper gives a satellite cluster simulation example to further demonstrate the topology control strategy used in the actual workflow. Additonally, some analysis about the simulation results are also discussed. In the discussion part, this paper involves two other usual topology control algorithms for comparison, MST and ${\mathrm{FlYG}}_{\pi /3}$. MST is a centralized algorithm to get a subset of the edges of a connected, edge-weighted undirected graph that connects all the nodes together. And it can be used to generate a spanning tree of minimum energy cost in MWSNs [21]. ${\mathrm{FlYG}}_{\pi /3}$ is a 3-dimensional Yao-based distributed algorithm, which uses identical cones with a top angle of π/3 to partition the transmission ball and to connect nodes in each partition [24]. The generated network topology of ${\mathrm{FlYG}}_{\pi /3}$ has the properties of bounded degree and energy efficiency.

This paper establishes a satellite cluster scenario with 16 satellites, including two EOS (Sat1, Sat2), two GSC (Sat15, Sat16), and 12 DRS (Sat3 -14). This paper supposes an EO mission that Sat1 and Sat2 send the observed image data to Sat 15 and Sat16. The satellite cluster scenario is tested with OMNET++ 5.0 [37] and the detailed simulation parameters are listed in Table 1.

**Figure 6:**
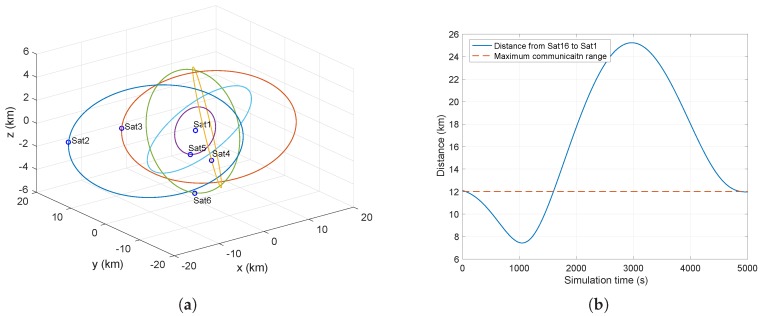
Illustration of the satellite cluster simulation scenario showing the trajectories of Sat2-6 moving with Sat1 (**a**) and distance varying with time from Sat16 to Sat1 (**b**).

Let Sat1 be the reference center of the relative motion coordinate system. Then, the rest can be described as running at a range of (0, 30 km) with Sat1. The main parameters of Sat1 are listed in Table 2. Figure 6a shows part of the satellite runs with Sat1 in the cluster. Figure 6b shows the periodic variation of distance from Sat16 to Sat1, where R=12km is the maximum communication range.

Before providing the experimental results regarding the topology lifetime and packet loss rate, we first observe the actual topologies at time t=0, for one simulated satellite cluster network using different topology control algorithms, including the proposed topology control strategy. Six figures are given here.

**Figure 7:**
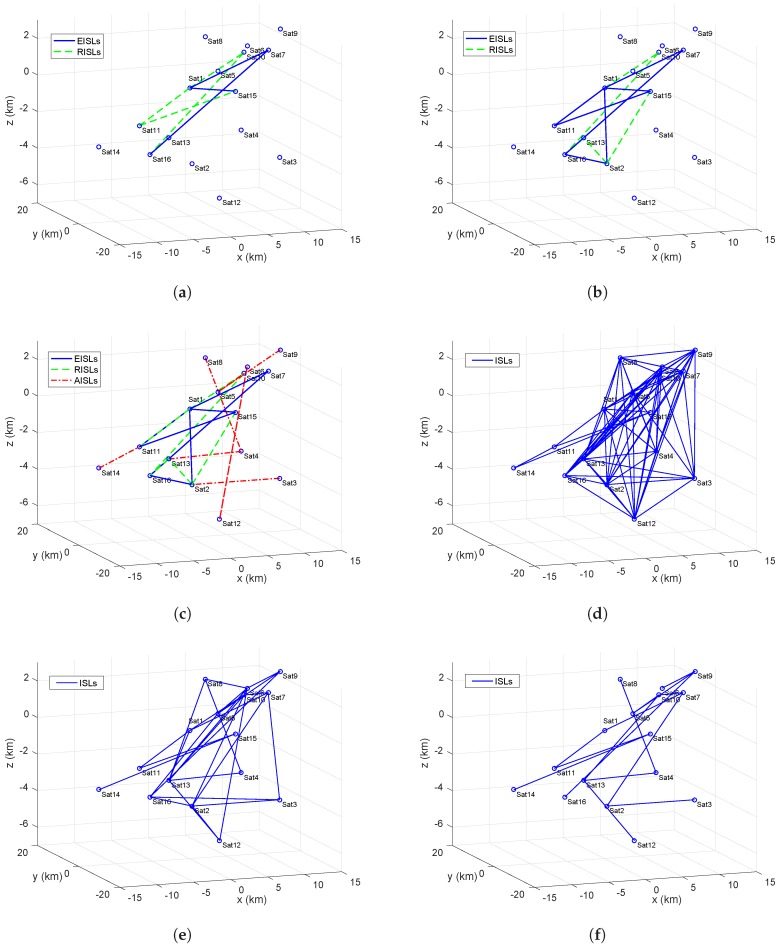
Network topologies of the satellite cluster with different topology control settings. (**a**) After applying Reliability-Assured Topology (RAT) algorithm Sub-step (1); (**b**) After applying RAT algorithm Sub-step (2); (**c**) After applying RAT algorithm Sub-step (3); (**d**) Without topology control; (**e**) After applying topology control algorithm ${\mathrm{FlYG}}_{2\pi /3}$; (**f**) After applying topology control algorithm minimum spanning tree (MST).

(1) Figure 7a shows the network topology after applying RAT algorithm Step 1.1–1.3, where the solid links (blue) denote the essential inter-satellite links (EISLs) and the dashed links (green) are the redundant inter-satellite links (RISLs). It is the key topology for Sat1 sending data to Sat15 and 16.
(2) Figure 7b shows the network topology after applying the RAT algorithm Step 1.4 and it adds the other key topology of Sat2 transmitting data to Sat15 and 16 in Figure 4a.
(3) Figure 7c is the final topology after applying the RAT algorithm, where the dot-dash links (red) are the additional inter-satellite links (AISLs).
(4) Figure 7d shows the origin of the physical topology without topology control. All nodes communicate with the maximal transmission range.
(5) Figure 7e,f represent the network topologies after using the ${\mathrm{FlYG}}_{2\pi /3}$ and MST algorithms.

First, this paper discusses two kinds of results, topology changing times and average topology lifetime, which can both reflect the network stability. Once the network topology changes, it may lead to the recalculating of routing paths with a result of increasing the end-to-end transmission delay. Thus, the lower topology changing times means the more stable the data transmission condition. Moreover, the average topology lifetime directly influences the amount of transmitted data in one topology lifetime.

**Figure 8:**
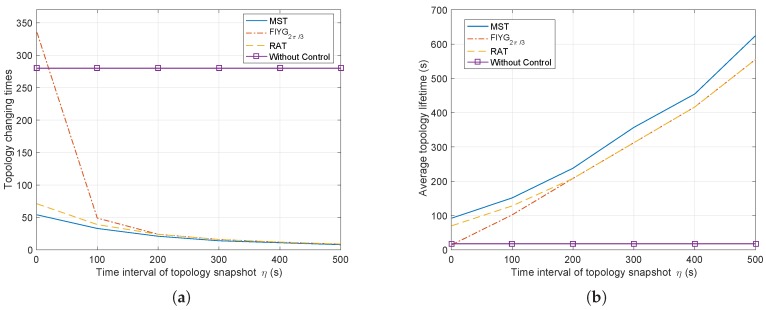
Topology changing results from three topology control algorithms (RAT, ${\mathrm{FlYG}}_{2\pi /3}$, MST) showing total topology changing times (**a**) and the average topology lifetime (**b**).

Figure 8a shows the network topology changing times varying with the time intervals of the snapshot. It is obvious that all of the three topology control algorithms show decreasing trends, and they finally trend to the same when η=500 s. The solid line with squares represents the simulation condition without any topology control algorithm, which serves as a contrast to the others. For most situations, it can be seen that when the network works without control, its topology changes as many as 280 times, much higher than the other three. When η∈(0,100), the results from ${\mathrm{FlYG}}_{2\pi /3}$ drops quickly from 335 to 49, also changing much more than RAT at around 40 times. The RAT results vary from 54 times to 20 times, and after the interval *η* increases to greater than 200 s, it changes little, about 15 times. Thus, compared to the without control and ${\mathrm{FlYG}}_{2\pi /3}$ scenarios, the network with RAT or MST make it much simpler for the upper communication protocol layers, like routing protocols, to keep the data transmitting paths stable, because of the fewer topology changes.

Figure 8b presents the average topology lifetime from the three different topology control algorithms (MST, ${\mathrm{FlYG}}_{2\pi /3}$, and RAT), and also without control. It is evident that the results from the without control condition have nothing to do with the time interval *η* and holds a constant value, 17.9 s. The other three algorithms show an increasing trend, with MST having the longest average topology lifetime in all situations. In addition, after the interval *η* increases to greater than 200 s, the results from RAT and ${\mathrm{FlYG}}_{2\pi /3}$ trend to be the same. Thus, it can be summarized that the topology control algorithm could greatly increase the average topology lifetime as the time interval grows, which would help the network to transmit more data due to the longer average topology lifetime.

In the meantime, this paper analyzes the total received packets and average packet loss rate which can be calculated through the following equation:

(18) $$Average\;packet\;loss\;rate=\frac{P_{send}\left(Sat1\right)+P_{send}\left(Sat2\right)-P_{received}\left(Sat15\right)-P_{received}\left(Sat16\right)}{(P_{send}\left(Sat1\right)+P_{send}\left(Sat2\right))t}$$

where $P_{send}\left(Sat1\right)$ is the amount of generated packets at Sat1, $P_{send}\left(Sat2\right)$ is the amount of generated packets at Sat2, $P_{received}\left(Sat15\right)$ is the amount of received packets at Sat15 from Sat1, $P_{received}\left(Sat16\right)$ is the amount of received packets at Sat16 from Sat2, and *t* is the simulation time. Both of these can directly affect the network performance in terms of the reliability of the topology control algorithms. It is clear that the lower the average packet loss rate means the better the algorithm performs in the network. Furthermore, the variation on the average packet loss rate gives the trends of the actual state of the network ISLs.

**Figure 9:**
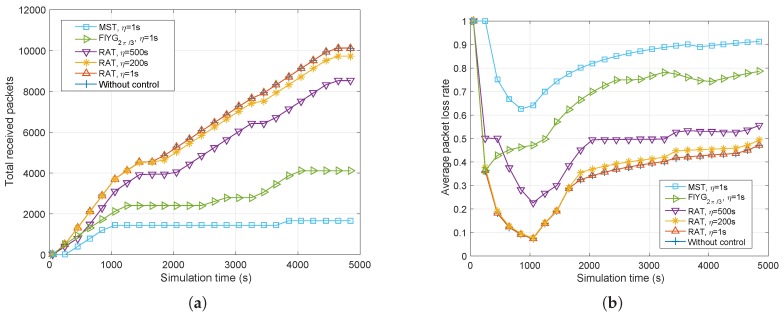
Results from three topology control algorithms (RAT, ${\mathrm{FlYG}}_{2\pi /3}$, MST) showing total received packets (**a**) and average packet loss rate (**b**).

Figure 9a shows the total packets received varying with the simulation time. When the network works with MST, the received packets increase slowly, especially after t>1000s. The network with ${\mathrm{FlYG}}_{2\pi /3}$ performs slightly better compared to MST. Comparing the situation without control with RAT (η=1s), it is clear that the results of two conditions are nearly the same. The without control scenario receives a total of 10,125 packets, while RAT (η=1s) receives 10,123 packets with just two packets left. This means that the topology with RAT (η=1s) maintains the most reliable ISLs compared to other algorithms. Additionally, when *η* in RAT increases, the performance of received packets goes down, but it also performs better than ${\mathrm{FlYG}}_{2\pi /3}$ and MST. Furthermore, when the simulation runs at time interval [1000,2000], all of the algorithms show the interruption of received data increasing, the reason being that the topology during that time changes so rapidly that the routing protocol on every satellite cannot find possible routing paths on time.

Figure 9b shows the average packet loss rate compared to the simulation time. It is obvious that MST performs the worst, with up to 90% packet loss, and ${\mathrm{FlYG}}_{2\pi /3}$ comes behind, with nearly 80%. Additionally, RAT (η=1s) presents the best average packet loss rate with a small difference to the condition without control. Although RAT (η=200s) shows slightly worse performance compared to RAT (η=1s), its topology changes approximately 62% less, which helps create fewer topology control messages in the network. It can be summarized that RAT performs better at adapting to changing network dynamics, resulting in a lower average packet loss rate.

Finally, it can be concluded in this simulation that ${\mathrm{FlYG}}_{2\pi /3}$ shows mediocre performance without any advantages. MST shows the fewest topology changing times and the longest average topology lifetime, but it performs the worst in terms of the average packet loss rate, which is up to 90%. Although RAT (η=1s) shows slightly worse performance compared to MST in average topology lifetime, it behaves the best with about 50% average packet loss rate. However, when η=1s, the topology changes frequently, which brings forward urgent demands for high accuracy of network synchronization and the satellite orbit dynamic model. And, RAT (η=200s) not only guarantees a proper average packet loss rate, it also makes it easier to implement in this satellite cluster network with EO missions.

## 6. Conclusions and Future Work

This paper studied the topology control problem in a satellite cluster network with EO missions by applying the link and network reliability metric. The motivation was to make the network more reliable by analyzing the proposed EO missions’ and ISLs’ reliability model. Then, this paper proposed the RAT algorithm and its corresponding topology control strategy to maximize the stability of data transmission in the satellite cluster network. Compared with most existing approaches for satellite cluster networks, where neither the periodicity of SCNs nor the predictability is adopted, the RAT uses both. The simulation results validated the theoretical analysis and effectiveness of the RAT algorithm. It showed that the RAT algorithm behaves the best of 50% average packet loss rate, compared to ${\mathrm{FlYG}}_{2\pi /3}$ of 80% and MST of 90%.

Although the assumptions stated in Section 2 are widely used in existing topology algorithms, some of them may not be practical. Our future work will focus on how to relax these constraints (e.g., satellites are homogeneous) for RAT algorithm so as to improve its practicality in actual applications. Furthermore, to make inter-satellite data transmission more practical in the future, it not only requires research on the topology control strategy, but also on the routing algorithms for the SCNs.

## Figures and Tables

**Table 1:** Main parameters of satellites in communication.

Parameters	Values
ISLs bandwidth	10 Mb/s
ISLs queue type	FIFO
ISLs queue length	100 Packets
Simulation duration	5000 s
Size of packet	512 Bytes
Terminal bitrate	8 Mbps
Maximum communication range	12 km

**Table 2:** Orbit parameters of Sat1.

Orbit Elements	Values
Semi-major Axis	6778.14 km
Eccentricity	0
Inclination	97.0346 deg
Argument of Perigee	0 deg
RAAN	279.066 deg
True Anomaly	0 deg
